# MHC associations of ankylosing spondylitis in East Asians are complex and involve non-HLA-B27 HLA contributions

**DOI:** 10.1186/s13075-020-02148-5

**Published:** 2020-04-09

**Authors:** Geng Wang, Tae-Hwan Kim, Zhixiu Li, Adrian Cortes, Kwangwoo Kim, So-Young Bang, Paul Leo, Matthew A. Brown, Huji Xu

**Affiliations:** 1 Department of Rheumatology and Immunology, Shanghai Changzheng Hospital, Second Military Medical University, Shanghai, China; 2 University of Queensland Diamantina Institute, University of Queensland, Brisbane, Australia; 3 Department of Biology, Kyung Hee University, Seoul, Republic of Korea; 4 Translational Genomics Group, Institute of Health and Biomedical Innovation, Queensland University of Technology, Translational Research Institute, Brisbane, Australia; 5 Wellcome Trust Centre for Human Genetics, University of Oxford, Oxford, UK; 6 Department of Rheumatology, Hanyang University Hospital for Rheumatic Diseases, Seoul, Republic of Korea; 7 Guy’s & St Thomas’ NHS Foundation Trust and King’s College London NIHR Biomedical Research Centre, London, England; 8 Beijing Tsinghua Changgung Hospital, School of Clinical Medicine, Tsinghua University, Beijing, 100084 China; 9 Peking-Tsinghua Center for Life Sciences, Tsinghua University, Beijing, China

**Keywords:** Ankylosing spondylitis, HLA, Association

## Abstract

**Background:**

The association of HLA-B*27 with AS is amongst the strongest of any known association of a common variant with any human disease. Nonetheless, there is strong evidence indicating that other *HLA-B* alleles are involved in the disease. European ethnicity studies have demonstrated risk associations with HLA-B*40 and multiple other HLA-B, HLA-A, and HLA class II alleles, and demonstrated that in that ethnic group, the amino acid sequence at position 97 in HLA-B is the key determinant of HLA associations with AS. A recent study in Korean AS cases and controls additionally identified association at HLA-C*15:02. In the current study, we examined the MHC associations of AS in an expanded East Asian cohort.

**Methods:**

A total of 1637 Chinese, Taiwanese, and Korean AS cases meeting the modified New York Criteria for AS, and 1589 ethnically matched controls, were genotyped with the Illumina Immunochip, including a dense coverage of the MHC region. HLA genotypes and amino acid composition were imputed using the SNP2HLA programme using the Han-MHC reference panel based on the data of Han Chinese subjects (*n* = 9689), and association tested using logistic regression controlling for population stratification effects.

**Results:**

A strong association was seen with HLA-B*27 (odds ratio (OR) = 205.3, *P* = 5.76 × 10^−244^). Controlling for this association, the strongest risk association is seen with HLA-C*15 at genome-wide significant level (OR = 7.62, *P* = 9.30 × 10^−19^), and confirmed association is also seen with HLA-B*40 at suggestive level (OR = 1.65, *P* = 2.54 × 10^−4^). At amino acid level, the strongest association seen in uncontrolled analysis was with histidine at position 114 in HLA-B (*P* = 7.24 × 10^−241^), but conditional analyses suggest that the primary amino acid associations are with lysine at position 70 and asparagine at position 97. Restriction of the *ERAP1* association with HLA-B27-positive AS, previously reported in European subjects, was confirmed in East Asians.

**Conclusions:**

This study confirms in East Asians that the HLA associations of AS are multiple, including previously reported associations at HLA-B*27, HLA-B*40, and HLA-C*15, as well as novel association with HLA-DQB1*04. The HLA-B associations are driven by the amino acids at positions 70 and 97, in the B pocket of HLA-B.

## Background

Ankylosing spondylitis (AS) is a highly heritable rheumatic disease characteristically causing chronic inflammation of the spine and sacroiliac joints, as well as in some patients affecting the peripheral joints, the anterior uvea, and less commonly other organs. The worldwide distribution of AS is closely related to the prevalence of HLA-B*27, although the underlying mechanism remains unclear. Whilst the HLA-B*27 allele is found in approximately 85% of patients, there is strong evidence indicating that other *HLA-B* alleles and MHC genes are involved in the disease, as well as non-MHC loci.

Direct genotyping studies in European case-control cohorts have demonstrated risk associations consistently with HLA-B*40 and variably reported associations with multiple other HLA-B, HLA-A, and HLA class II alleles. The development of accurate HLA imputation methods from single nucleotide polymorphism (SNP) microarray data has enabled far larger case-control studies to be performed, with, for the first time, proper control for population stratification effects. Using this approach and studying 22,647 AS cases and controls of European descent, Cortes et al. demonstrated that the amino acid sequence of HLA-B at position 97, in the epitope-binding groove, is the key determinant of HLA associations with AS. After controlling for the associated alleles in *HLA-B*, independent associations with variants in the *HLA-A*, *HLA-DPB1*, and *HLA-DRB1* loci were observed [1].

Differences in HLA-B*27 subtype distributions between Asian and European descent populations have been well reported, and further non-HLA-B*27 HLA class I associations in East Asian AS have been reported. Also using HLA imputation methods, a study in 654 Korean cases of AS and 3166 controls additionally identified association at HLA-C*15:02 [2]. Additionally, using direct genotyping in 360 Han Chinese AS cases and 350 controls with no genomic control for population stratification, risk association of HLA-B*40 and protective association of HLA-B*07 have been demonstrated [3].

In this study, using HLA imputation methods, we analyse the associations of AS with major histocompatibility complex (MHC) polymorphisms to identify functional and potentially causal variants using a large cohort of East Asian ancestry AS cases and controls [4]. In addition to our primary analysis of this cohort, we perform fine mapping of the MHC region with imputation of SNPs, HLA class I and II classical alleles, and amino acid residues within the classical HLA proteins. In addition to HLA-B*27, we identify further HLA-B and other HLA class I and II alleles associated with AS.

## Methods

### Subjects and SNP data

A total of 1637 Chinese, Taiwanese, and Korean AS cases meeting the modified New York Criteria for AS [5] as confirmed by qualified rheumatologists, and 1589 ethnically matched controls (Table 1), were genotyped with the customised SNP array (Illumina Immunochip [6]), including a dense coverage of the MHC region. Cohort descriptions and genotyping protocols are as previously reported [4]. By standard quality control procedures, SNPs with a minor allele frequency of at least 1% (MAF > 0.01), call rates of ≥ 0.98, and *P* values in Hardy-Weinberg disequilibrium tests ≤ 10^−7^ were analysed in this study. To confirm ethnicity, we performed a continental principal components analysis (PCA), merging the study genotype data available from 51 available populations genotyped by Illumina 650Y from the Human Genome Diversity Panel (HGDP-CEPH) [7]. Cases or controls lying more than 6 standard deviations from the population mean on principal components (PCs) 1–10 were then excluded.

**Table 1:** Demographic summary of the study cohort

	Cases	Controls	Total	Percentage
Chinese	764	683	1447	45
Taiwanese	214	179	393	12
Korean	659	727	1386	43
Total	1637	1589	3226	–

### HLA imputation and association analysis

We conducted a 2-step imputation. We densely imputed SNPs across the MHC using the Michigan Imputation Server [8] and the 1000 Genomes Phase 3 reference dataset (26 populations across the world), then further using the Han-MHC reference panel [9], to ensure maximum SNP coverage to enable accurate imputation of HLA-B alleles, including of particular interest, HLA-B27. Using this SNP data and the Han Chinese reference panel (*N* = 9869), the programme SNP2HLA was used to impute the classic HLA alleles and amino acid residues of the 8 HLA genes (*HLA-A*, *HLA-B*, *HLA-C*, *HLA-DPB1*, *HLA-DQB1*, *HLA-DRB1*, *HLA-DPA1*, *HLA-DQA1*) in a total of 3007 East Asian subjects. In the output file of SNP2HLA, imputed classical HLA alleles and HLA protein amino acid positions were defined as binary markers coding the presence or absence of the allele or residue being tested, and each different allele or residue was tested as a biallelic position. Association with AS was then tested using logistic regression function in PLINK [10] by including all allele/residues/SNP conditioning on 10 principal components to control for population stratification effects. We then performed conditional analysis repeatedly in an iterative fashion by adding the dosage of HLA-B*27 allele and other significant alleles/residues/SNPs as covariates until no significant allele/residue/SNP was observed. Only *HLA* alleles or amino acids with imputation information scores > 0.5 were considered. All results are presented unadjusted for multiple testing.

## Results

PCA indicated that all study subjects were ethnically East Asian (Supplementary Figure 1). The genomic inflation factor calculated using a set of 1767 negative control SNPs in regions included on Immunochip for studies of reading and writing disabilities, psychosis, and schizophrenia was 1.03 (lambda (1000) = 1.02). No evidence of statistical inflation is seen in the Q-Q plot (Supplementary Figure 2). After quality control and imputation, 15,748 SNPs across the MHC (from 25 to 35 Mb, hg18) were available for analysis in 1482 cases and 1512 controls. Imputed HLA-B allele frequencies amongst controls in the current study were not significantly different from those in previously reported directly genotyped studies (*P* > 0.05), confirming the high accuracy of HLA imputation, particularly at a two-digit resolution [3].

### HLA-B associations

**Fig. 1:**
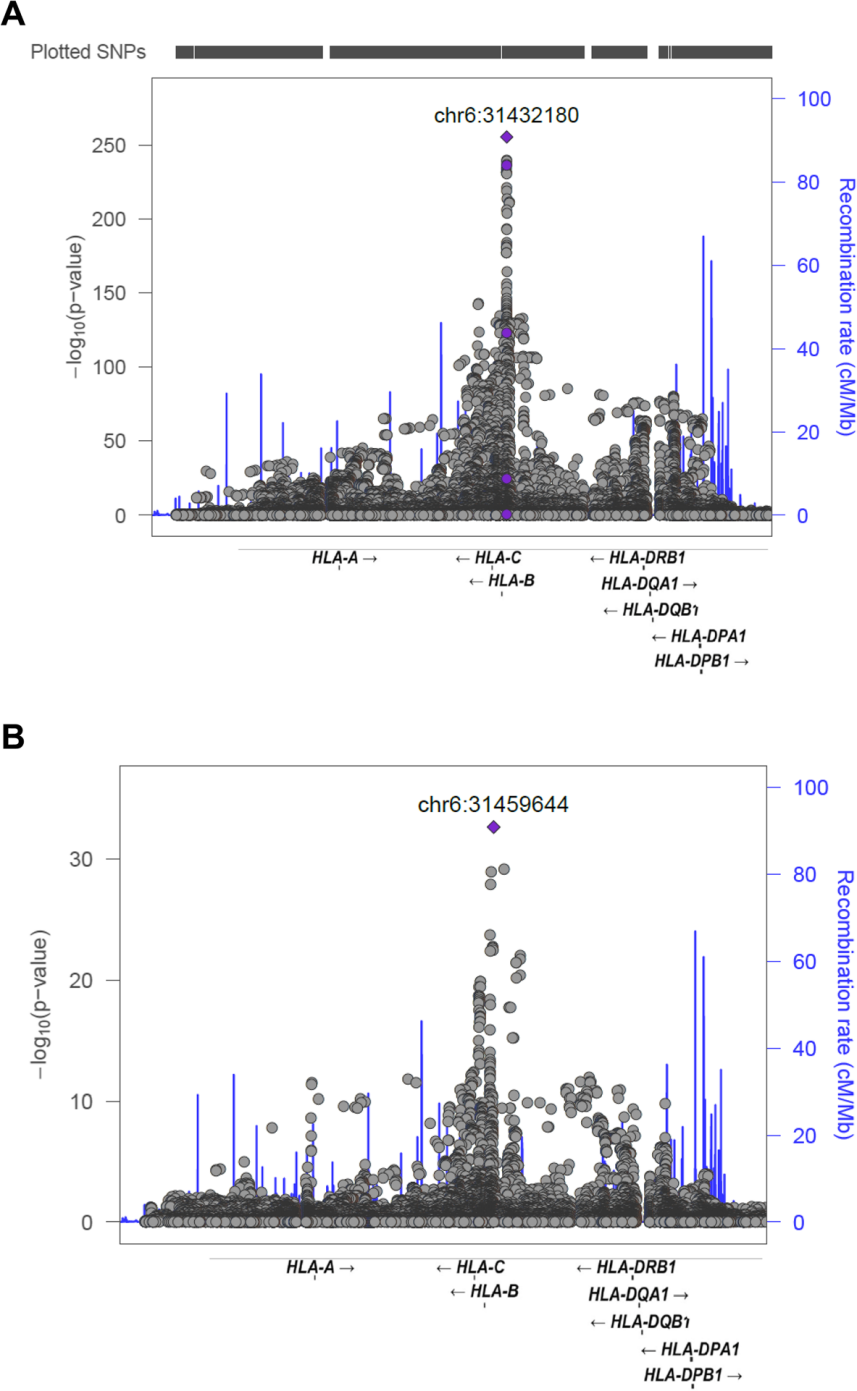
AS susceptibility associations in the MHC region. Association plots for the extended major histocompatibility complex region. Significance levels of each marker SNPs were calculated by using logistic regression of imputed dosage files and plotted according to chromosomal locations (based on hg19). **a** Top association was identified at HLA-B27. **b** There were residual signals with SNPs near HLA-A, HLA-B, HLA-C, and HLA class II alleles conditioning on the HLA-B*27

The strongest SNP association with AS observed was a missense variant of *HLA-B*, rs1071652 (SNP-B*31432180_CG, odds ratio (OR) = 180, *P* = 4.45 × 10^−256^, Fig. 1). The previously reported East Asian HLA-B*27 tagSNP rs13202464 (31452562) [11] was also found significantly associated with AS (OR = 58.73, *P* = 1.92 × 10^−211^). Controlling for rs1071652, residual association is seen with HLA-B*27 (4.48 × 10^−22^) and SNP rs41553720 (SNP-B*31432843_A, *P* = 3.87 × 10^−31^), indicating that combinations of SNPs are currently required to tag HLA-B*27 in East Asian populations, in contrast to the situation in European descent populations [12].

After SNP imputation in the MHC region, the expected strong association was observed with HLA-B*27 (odds ratio (OR) = 205, *P* = 5.76 × 10^−244^, Table 2). Controlling for the HLA-B*27 association and studying other HLA-B alleles, risk association is seen with HLA-B*40 at suggestive level (OR = 1.65, *P* = 2.54 × 10^−4^). Controlling for both HLA-B*27 and HLA-B*40, no association was observed in the 2-digit HLA-B allele with MAF > 1% (only rare alleles HLA-B*53 and HLA-B*38 were associated at suggestive level; *P* values were 2.9 × 10^−4^ and 3.3 × 10^−4^, respectively).

**Table 2:** Association of HLA alleles with susceptibility to ankylosing spondylitis (*P* < 0.05 in conditional analysis on *HLA-B27*)

SNP	FRQ	OR	*P*	*P*_con_B27	*P*_con_B27_B40
2-digit HLA alleles					
HLA-C*15	0.04	1.01	0.97	9.30 × 10^−19^	9.88 × 10^−17^
HLA-DQB1*04	0.07	0.96	0.68	5.98 × 10^−7^	1.86 × 10^−6^
HLA-DRB1*13	0.07	0.37	6.28 × 10^−15^	2.53 × 10^−4^	7.85 × 10^−4^
HLA-B*40	0.12	0.58	2.63 × 10^−11^	2.54 × 10^−4^	NA
HLA-C*03	0.20	0.43	9.19 × 10^−33^	6.08 × 10^−4^	9.92 × 10^−5^
HLA-DQB1*06	0.22	0.52	1.50 × 10^−20^	6.32 × 10^−4^	1.25 × 10^−3^
HLA-B*53	0.00	0.62	0.46	7.33 × 10^−4^	2.87 × 10^−4^
HLA-B*38	0.01	2.10	0.041	9.55 × 10^−4^	3.35 × 10^−4^
HLA-C*05	0.01	0.25	2.75 × 10^−3^	1.77 × 10^−3^	2.68 × 10^−3^
HLA-B*15	0.10	0.40	1.13 × 10^−21^	0.021	0.082
HLA-DRB1*04	0.15	1.09	0.26	0.028	0.032
HLA-DPB1*45	0.00	0.98	0.97	0.029	0.025
HLA-DQA1*06	0.06	4.09	3.05 × 10^−17^	0.048	0.035
4-digit HLA alleles					
HLA-C*1502	0.03	1.15	0.37	6.78 × 10^−23^	4.10 × 10^−20^
HLA-B*4002	0.03	0.79	0.16	9.55 × 10^−12^	6.54 × 10^−8^
HLA-DRB1*0405	0.06	0.93	0.51	2.30 × 10^−7^	1.06 × 10^−6^
HLA-DQB1*0401	0.05	0.90	0.45	5.05 × 10^−6^	1.54 × 10^−5^
HLA-B*4005	0.00	1.72	0.55	4.21 × 10^−4^	2.09 × 10^−3^
HLA-B*5301	0.00	0.62	0.46	7.20 × 10^−4^	2.82 × 10^−4^
HLA-DRB1*1302	0.05	0.37	5.63 × 10^−13^	1.13 × 10^−3^	2.88 × 10^−3^
HLA-B*3802	0.01	2.29	0.036	2.16 × 10^−3^	1.01 × 10^−3^
HLA-C*0501	0.00	0.38	0.054	3.98 × 10^−3^	5.60 × 10^−3^
HLA-DQA1*0102	0.27	0.36	3.45 × 10^−30^	4.68 × 10^−3^	0.013
HLA-B*4446	0.03	0.21	5.69 × 10^−14^	6.15 × 10^−3^	0.012
HLA-C*0304	0.08	0.53	1.72 × 10^−9^	0.012	6.95 × 10^−5^
HLA-DRB1*1501	0.08	0.56	5.36 × 10^−8^	0.022	0.024
HLA-B*4402	0.00	0.30	0.13	0.027	0.033
HLA-DPB1*4501	0.00	0.97	0.96	0.029	0.024
HLA-DQB1*0603	0.01	0.41	2.55 × 10^−3^	0.036	0.055
HLA-B*1501	0.04	0.24	2.94 × 10^−15^	0.047	0.11
HLA-DQA1*0601	0.06	4.09	3.04 × 10^−17^	0.048	0.035
HLA-B*5502	0.02	0.28	9.64 × 10^−6^	0.050	0.10

*OR* odds ratio in unconditional analysis, *FRQ* allele frequency in controls, *P P* value in unconditional analysis, *P_con_B27 P* value in conditional analysis controlling HLA-B*27, *P_con_B27_B40 P* value in conditional analysis controlling HLA-B*27 and HLA-B*40

At the amino acid level, the strongest association seen in the uncontrolled analysis was with histidine at position 114 in HLA-B (*P* = 7.24 × 10^−241^), followed by multiple HLA-B amino acids including lysine at 70 (*P* = 1.49 × 10^−237^) and asparagine 97 (*P* = 2.51 × 10^−237^) (Table 3). Asparagine 97 and histidine 114 were previously reported to be the main amino acid determining HLA-B associations with AS in European descent and Korean populations, respectively [1, 2].

**Table 3:** Association of amino acid residues in HLA-B with susceptibility to ankylosing spondylitis (*P* < 10^−100^ in unconditional analysis)

Position	AA	FRQ	OR	*P*	*P*_con_B27	*P*_con_B27_B40
114	H	0.24	232.78	7.24 × 10^−241^	1.86 × 10^−8^	1.18 × 10^−8^
70	K	0.25	312.90	1.49 × 10^−237^	1.22 × 10^−40^	1.35 × 10^−38^
97	N	0.24	255.79	2.51 × 10^−237^	1.08 × 10^−13^	3.49 × 10^−13^
67	C	0.30	62.52	8.05 × 10^−232^	6.91 × 10^−17^	6.89 × 10^−18^
116	D	0.29	57.93	2.38 × 10^−221^	0.15	0.061
80	T	0.31	42.93	1.99 × 10^−220^	2.82 × 10^−20^	1.27 × 10^−21^
113	Y	0.32	25.09	5.03 × 10^−194^	0.33	0.12
9	H	0.39	16.96	2.20 × 10^−154^	1.00 × 10^−11^	3.64 × 10^−14^
45	E	0.39	14.84	5.35 × 10^−153^	2.41 × 10^−9^	3.46 × 10^−11^
9	Y	0.39	16.08	1.75 × 10^−151^	3.24 × 10^−11^	2.68 × 10^−12^
69	A	0.40	11.19	1.81 × 10^−146^	8.20 × 10^−7^	1.13 × 10^−8^
11	S	0.41	13.63	4.25 × 10^−145^	8.62 × 10^−14^	1.66 × 10^−11^
83	R	0.42	13.77	8.00 × 10^−145^	1.76 × 10^−14^	7.46 × 10^−18^
12	V	0.41	12.47	9.64 × 10^−143^	6.01 × 10^−13^	9.44 × 10^−11^
82	L	0.44	10.47	2.64 × 10^−140^	8.24 × 10^−12^	3.80 × 10^−15^
32	L	0.41	11.00	7.76 × 10^−137^	3.06 × 10^−7^	1.38 × 10^−4^
80	N	0.45	8.89	3.98 × 10^−135^	8.83 × 10^−13^	1.49 × 10^−16^
24	T	0.44	9.12	2.58 × 10^−134^	3.08 × 10^−9^	8.53 × 10^−7^
77	D	0.14	27.62	5.68 × 10^−128^	0.39	0.39
74	D	0.43	7.41	6.83 × 10^−126^	7.02 × 10^−3^	2.97 × 10^−4^
163	E	0.46	6.58	1.53 × 10^−121^	2.06 × 10^−3^	0.38
69	T	0.46	5.72	4.97 × 10^−117^	5.01 × 10^−4^	5.62 × 10^−6^
− 16	L	0.48	5.77	4.58 × 10^−116^	1.76 × 10^−6^	1.28 × 10^−5^
− 16	V	0.48	5.69	2.93 × 10^−115^	1.66 × 10^−6^	1.17 × 10^−5^
71	A	0.47	5.49	5.24 × 10^−114^	1.94 × 10^−3^	3.15 × 10^−5^
70	N	0.47	5.48	5.55 × 10^−114^	1.96 × 10^−3^	3.19 × 10^−5^
97	R	0.54	5.13	9.25 × 10^−109^	7.34 × 10^−7^	2.13 × 10^−6^
24	A	0.43	0.23	6.90 × 10^−103^	3.58 × 10^−8^	1.14 × 10^−5^

*OR* odds ratio in unconditional analysis, *P P* value in unconditional analysis, *P_con_B27 P* value in conditional analysis controlling HLA-B*27, *P_con_B27_B40 P* value in conditional analysis controlling HLA-B*27 and HLA-B*40. NS = *P > 0.05*

Conditional analyses for these individual amino acids and their combinations reveal that only the association of histidine 114 can be attenuated by conditioning on asparagine 97 (Table 4). No other individual amino acid explains the association of the other amino acids. For HLA-B alleles, both combinations of lysine 70 + asparagine 97 and lysine 70 + histidine 114, but not asparagine 97 + histidine 114, controlled for association with any other 2-digit HLA-B allele (*P* > 0.0017, correcting for 30 2-digit HLA-B alleles tested). Controlling for all of lysine 70, asparagine 97, and histidine 114, the strongest HLA amino acid association remains with several positions including HLA-B position 97 (serine, found on HLA-B*7, *8, *15, *2707, *40, *41, *48; OR = 2.14, *P* = 2.14 × 10^−5^).

**Table 4:** Conditional analysis *P* values of HLA-B amino acid residues. Significance for association of lysine 70 (70K), asparagine 97 (97N), and histidine 114 (114H) is given in columns, either in unconditional analysis or conditioning on specific amino acid positions (where no letter is given after the HLA-B amino acid position number) or for specific amino acids (where a letter is given after the HLA-B amino acid position number), either individually or in combinations. NS = *P* > 0.05

	70K	97N	114H	HLA-B*27
Unconditional	1.49 × 10^−237^	2.51 × 10^−237^	7.24 × 10^−241^	5.76 × 10^−244^
70	–	1.10 × 10^−6^	1.76 × 10^−6^	9.38 × 10^−7^
97	1.04 × 10^−28^	–	0.13	0.18
114	4.08 × 10^−37^	2.70 × 10^−8^	–	5.25 × 10^−6^
70 + 97	–	–	0.24	0.11
70 + 114	–	2.94 × 10^−1^	–	0.20
97 + 114	5.81 × 10^−28^	–	–	0.066
70 + 97 + 114	–	–	–	0.21
70K	–	6.15 × 10^−7^	1.04 × 10^−6^	4.83 × 10^−7^
97N	1.11 × 10^−31^	–	0.42	0.020
114H	1.70 × 10^−38^	2.02 × 10^−8^	–	2.47 × 10^−6^
70K + 97N	–	–	0.49	0.20
70K + 114H	–	0.30	–	0.18
97N + 114H	1.27 × 10^−31^	–	–	0.32
70K + 97N + 114H	–	–	–	0.28

Controlling for HLA-B*27 alone or in combination with HLA-B*40 did not fully control for the association of asparagine 97, lysine 70, or histidine 114 (*P* < 5 × 10^−8^ for both analyses, Table 3).

### Non-HLA-B susceptibility loci in the MHC

Considering HLA alleles other than HLA-B, several HLA-A, HLA-C, and HLA class II alleles showed significant associations (Table 2). Controlling for the HLA-B*27 association, independent risk association was confirmed with HLA-C*15 (OR = 2.13, *P* = 9.30 × 10^−19^) and HLA-C*1502 (OR = 7.62, *P* = 6.78 × 10^−23^), both associations at the amino acid level tagged by a leucine 116 in HLA-C (*P* = 6.61 × 10^−21^) located in the HLA-C epitope-binding groove. Stepwise conditional analyses on both HLA-B*27 and HLA-C*15 demonstrated significant associations with HLA-DQB1*04 (OR = 2.13, *P* = 7.91 × 10^−5^) after correcting for multiple comparisons (499 signals across MHC, as defined by regions with LD *r*^2^ < 0.2, Bonferroni correction threshold = 10^−4^). Conditioning on both HLA-B*27 and HLA-B*40, the association was confirmed with HLA-C*15 (OR = 4.97, *P* = 9.88 × 10^−17^) and HLA-DQB1*04 (OR = 2.42, *P* = 1.86 × 10^−6^).

### *ERAP1* variants in association with AS

The key *ERAP1* variant associated with AS is rs30187 (ccc-5-96150086-T-C, chr5:96150086[hg18], encoding K528R) [4, 13] (Table 5). It has previously been observed in European populations that the association with the variant rs30187 in the *ERAP1* locus is restricted to HLA-B*27-positive subjects, or HLA-B*40-positive, HLA-B27-negative subjects, consistent with epistatic interactions. Here, we investigated the possibility of interaction between the HLA-B*27 and HLA-B*40 alleles and the previously reported tag SNP of *ERAP1* locus (rs30187) [4]. When testing for interaction with the HLA-B*27 alleles, we found that rs30187-A risk allele increased the risk of disease in the strata where HLA-B*27 was present (OR = 1.29; *P* = 2.71 × 10^−6^) (Table 5), but no association was seen in HLA-B27-negative cases (OR = 1.06, *P* = 0.61). No evidence of interaction was observed between rs30187 and the HLA-B*40 allele, although the power to identify this was low as the number of HLA-B27-negative cases was low.

**Table 5:** Association analysis of rs30187 in samples positive and negative for HLA-B*27 and HLA-B*40. Odds ratios are given for the rs30187-risk A allele

Group (case/control)	OR	*P*
All (1482/1512)	1.26	9.00 × 10^−6^
HLA-B27+ cases vs all controls (1323/1512)	1.29	2.71 × 10^−6^
HLA-B27− cases vs all controls (159/1512)	1.06	0.61
HLA-B27+ cases and controls (1323/77)	1.40	0.044
HLA-B27− cases and controls (159/1435)	1.06	0.64
HLA-B27+ cases vs HLA-B27− cases (1323/159)	1.21	0.11
HLA-B27+/HLA-B40+ (207/11)	1.25	0.60
HLA-B27+/HLA-B40− (1116/66)	1.42	0.050
HLA-B27−/HLA-B40− (89/1027)	1.11	0.49
HLA-B27−/HLA-B40+ (70/408)	1.02	0.91

*OR* odds ratio in unconditional analysis, *P P* value in unconditional analysis, *SE* standard error of beta (log-odds) estimate, *CHR* chromosome

## Discussion

This study confirms that in East Asians, the primary MHC associations with AS are with HLA-B*27 and HLA-B*40, and confirms the risk association of HLA-C*1502 with the disease. The association of HLA-B*40 with AS has been convincingly demonstrated now in both European descent [1, 14–16] and East Asian studies [3, 17], using both direct genotyping- and imputation-based methods. HLA-B*4001 has also been shown to be associated with IgA nephropathy (OR = 1.34, *P* = 5.64 × 10^−7^) [18], a known though uncommon association of AS. The functional mechanism of association of this allele has been little studied. It does not share the lysine 70, asparagine 97, or histidine 114 residues found in most HLA-B*27 alleles. As with HLA-B*27, it is known to interact with AS-associated *ERAP1* variants to cause AS, suggesting that it is likely to operate by the same mechanism. Further studies to compare its properties with HLA-B27, such as its peptide-binding characteristics, folding rate, and whether it forms homodimers, are indicated to investigate its association further.

No protective association was seen with HLA-B*07 as has previously been reported in East Asians [3] and European descent cohorts [1, 15], although the allele frequency was very low and the study may not have had adequate power to detect any association with the allele (frequency = 0.024).

The study indicates that in East Asians, the key amino acid drivers of the HLA-B associations in AS are amino acid positions 70 and 97. These remain AS-associated controlling for any other HLA-B amino acid. HLA-B position 97 was previously shown in European descent cohorts to be the key amino acid association in the broad ethnicity, whereas in a Korean study, the association of histidine 114 could not be distinguished from associations with lysine 70 and asparagine 97 [2]. The difference in these findings may be explained by three key factors, sample size, ethnicity, and the reference haplotype dataset. Cortes et al.’s study of European descent subjects involved 9069 AS cases and 13578 controls, over seven times as many subjects as involved in the current study (1637 cases, 1589 controls) and nearly six times the number involved in the previous Korean study (654 cases, 3166 controls). Therefore, the European descent study had greater power, potentially explaining the absence of signal in the East Asian cohorts for some of the HLA-B allele and the HLA class II associations seen in the European dataset. The European descent study also has greater power in conditional analyses, potentially explaining the differences in results regarding the role of lysine 70, which remains positively associated with AS after conditioning on asparagine 97 in the current study, but not in the European descent dataset. The different studies have also used different reference haplotype datasets, potentially affecting the accuracy of the imputation data. Ethnic differences could also play a role through differences in HLA-B*27 subtypes or other HLA-B allele frequencies, particularly comparing the European descent and East Asian cohorts.

Both HLA-B amino acid residues 70 and 97 are found within the B pocket of the HLA-B peptide-binding groove. However, it has been noted that position 70 is tightly coupled with positions 67 and 97 and that position 70 hardly changes the peptide-binding repertoire, suggesting that position 70 is “hitch-hiking” along with positions 67 and 97 in their ability to change the peptide-binding repertoire [19]. Our study and the previous HLA amino acid imputation studies suggest that other amino acid positions in addition to 70 (like position 97 and 114) are also involved in HLA-B risk attribution. The association of these amino acids independent of other amino acids found in the HLA-B27 B pocket, and having controlled for HLA-B27, indicates that their effect on disease risk is partially independent of HLA-B27.

Although the HLA allele frequencies imputed in controls in this study closely match those reported by direct genotyping studies in Han Chinese [3], the accuracy of imputation in such studies is very dependent on the ethnic matching of the imputed and reference datasets. Whilst the Han-MHC reference dataset used here is of large size (*n* = 9689), the number of East Asian in 1000 Genomes Phase 3 (*n* = 524), which we used in the Michigan Imputation Server, is far smaller than the European dataset used in Cortes et al. (Type 1 Diabetes Genetics Consortium dataset, *n* = 5225) [1]. The smaller reference dataset size precluded imputation to four-digit levels and may have affected the accuracy of the imputation of low-frequency alleles in particular. As SNP-based HLA imputation is a highly efficient method enabling large-scale HLA association studies, there is a clear need for much larger publicly available HLA imputation reference datasets for Asian populations.

In this study, we have also confirmed the interaction between *ERAP1* and HLA-B*27, with association only observed of the key *ERAP1* variant, rs30187, only seen in HLA-B*27-positive individuals. This confirms the original finding in Europeans [1] and the previous finding in a case-only analysis of Taiwanese AS patients of different *ERAP1* genotypes in HLA-B*27-positive and HLA-B*27-negative cases [20]. We did not see an association of *ERAP1* variants in HLA-B*27-negative and HLA-B*40-positive individuals as previously reported [1], although the sample size was not large. The confirmation of the gene-gene interaction in an East Asian population increases the evidence that this is a true-positive interaction and is critical to AS pathogenesis.

## Conclusions

This study confirms that the HLA associations of AS are complex and that multiple non-HLA-B*27 alleles, including both HLA class I and likely HLA class II variants, also contribute to risk and protection from the disease. Further investigation of the mechanisms involved in these associations is likely to assist in determining the pathogenesis of this disease.

## Supplementary information

**Additional file 1.**

## Data Availability

Summary data for the datasets used are available at Harvard Dataverse (10.7910/DVN/NJ7XSO) and on request from the authors.

## Abbreviations

AS: Ankylosing spondylitis
HGDP-CEPH: Human Genome Diversity Panel
MHC: Major histocompatibility complex
MAF: Minor allele frequency
OR: Odds ratio
PCs: Principal components
PCA: Principal components analysis
SNP: Single nucleotide polymorphism
