# Supplementary material for: MHC associations of ankylosing spondylitis in East Asians are complex and involve non-HLA-B27 HLA contributions
Source: Arthritis Res Ther. 2020 Apr 9;22:74. doi: 10.1186/s13075-020-02148-5 (PMC7146985; doi:10.1186/s13075-020-02148-5)

Supplementary Figure 1: Continental PCA plot in all study samples merged with SNP data from 51 known ethnic groups genotyped by Omni-650 Beadchips. Colours and shapes represent study populations (see legends above), including AS samples (black dots), and controls (grey dots) in current study. The black square includes all study samples and red lines show 6 standard deviations from the population mean on principal components.


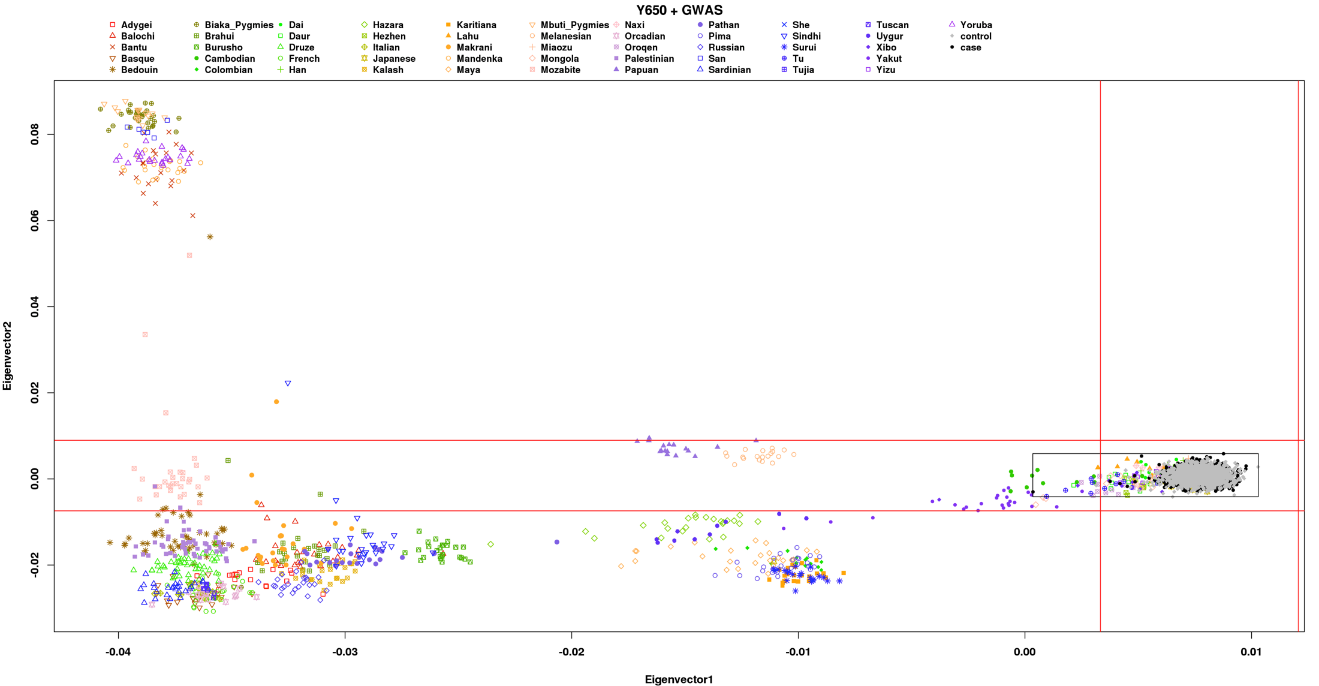


Supplementary Figure 2: Q-Q plots of association for negative control SNPs set

Observed versus expected association for a set of negative control SNPs in regions included on Immunochip for studies of reading and writing disabilities, psychosis and schizophrenia. For the analyses, the negative control SNP lists contained SNPs that were unlinked (r2 < 0.3) and common (MAF > 0.05) in the respective controls sample set. This resulted in a list of 1,767 SNPs for the East Asian ethnicity analysis. The genomic inflation factor in the negative control SNP list in the overall analysis was 1.03 (lambda(1000)=1.02).


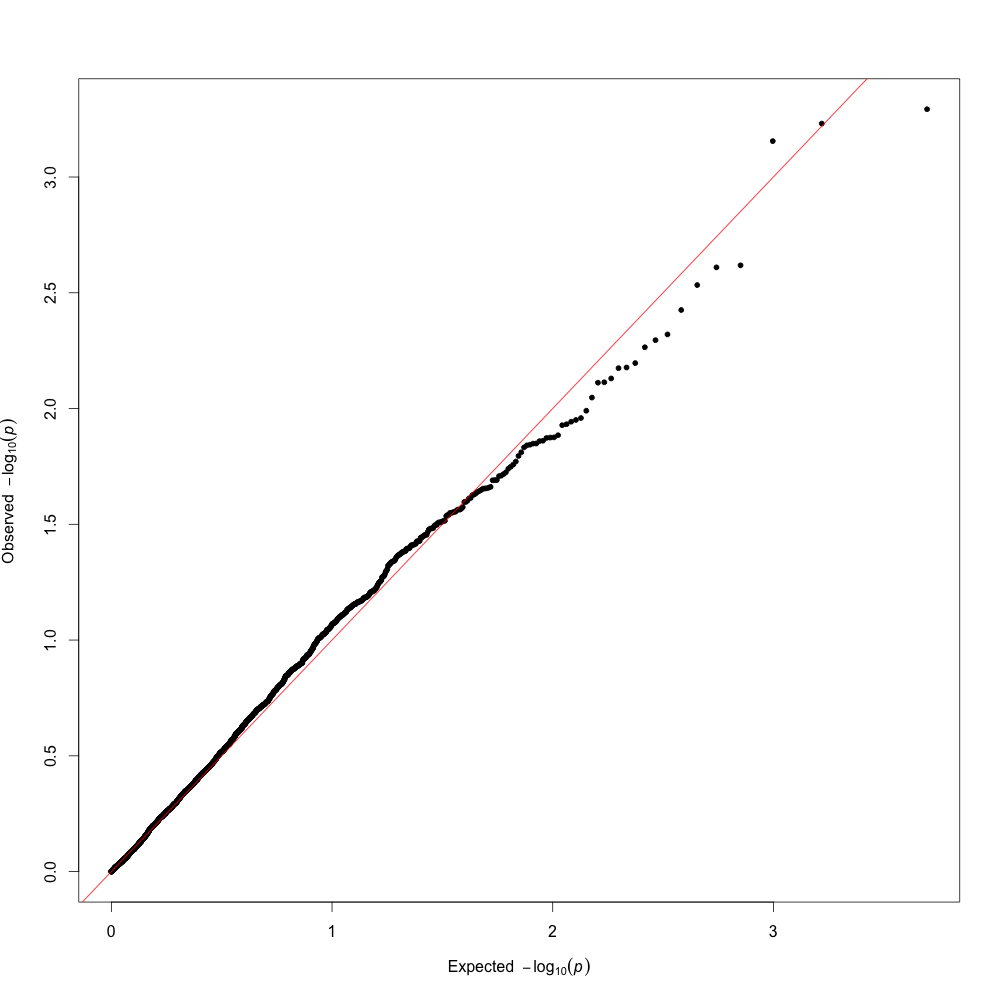

Supplement: Supplementary file 1 — Additional file 1. [file 13075_2020_2148_MOESM1_ESM.docx]
